# Defining the next generation of severe malaria treatment: a target product profile

**DOI:** 10.1186/s12936-024-04986-z

**Published:** 2024-06-05

**Authors:** Jane Achan, Aïssata Barry, Didier Leroy, George Kamara, Stephan Duparc, Wiweka Kaszubska, Preetam Gandhi, Bénédicte Buffet, Patrick Tshilab, Bernhards Ogutu, Terrie Taylor, Sanjeev Krishna, Naomi Richardson, Hanu Ramachandruni, Hans Rietveld

**Affiliations:** 1 Malaria Consortium, London, UK; 2 Centre National de Recherche et de Formation sur le Paludisme, Ouagadougou, Burkina Faso; 3 Medicines for Malaria Venture, Route de Pré-Bois 20, Post Box 1826, CH-1215 Geneva 15, Switzerland; 4 Médecins Sans Frontières, Magburaka District Hospital, Freetown, Sierra Leone; 5 Novartis, Basel, Switzerland; 6 Ministry of Health, Lusaka, Zambia; 7 Centre for Clinical Research, Kenya Medical Research Institute, Kisumu, Kenya; 8 Queen Elizabeth Central Hospital and Blantyre Malaria Project, Kamuzu University of Health Sciences, Blantyre, Malawi; 9 Institut Für Tropenmedizin, Eberhard Karls Universität Tübingen, and German Center for Infection Research (Dzif), Tübingen, Germany; 10 Centre de Recherches Médicales de Lambaréné (CERMEL), Lambaréné, Gabon; 11 Clinical Academic Group, Institute for Infection and Immunity, St. George’s University of London, London, UK; 12 St George’s University Hospitals NHS Foundation Trust, London, UK; 13 Magenta Communications Ltd, Abingdon, UK

**Keywords:** Severe malaria, Drug development, Translational medicine

## Abstract

**Background:**

Severe malaria is a life-threatening infection, particularly affecting children under the age of 5 years in Africa. Current treatment with parenteral artemisinin derivatives is highly efficacious. However, artemisinin partial resistance is widespread in Southeast Asia, resulting in delayed parasite clearance after therapy, and has emerged independently in South America, Oceania, and Africa. Hence, new treatments for severe malaria are needed, and it is prudent to define their characteristics now. This manuscript focuses on the target product profile (TPP) for new treatments for severe malaria. It also highlights preparedness when considering ways of protecting the utility of artemisinin-based therapies.

**Target product profile:**

Severe malaria treatments must be highly potent, with rapid onset of antiparasitic activity to clear the infection as quickly as possible to prevent complications. They should also have a low potential for drug resistance selection, given the high parasite burden in patients with severe malaria. Combination therapies are needed to deter resistance selection and dissemination. Partner drugs which are approved for uncomplicated malaria treatment would provide the most rapid development pathway for combinations, though new candidate molecules should be considered. Artemisinin combination approaches to severe malaria would extend the lifespan of current therapy, but ideally, completely novel, non-artemisinin-based combination therapies for severe malaria should be developed. These should be advanced to at least phase 2 clinical trials, enabling rapid progression to patient use should current treatment fail clinically. New drug combinations for severe malaria should be available as injectable formulations for rapid and effective treatment, or as rectal formulations for pre-referral intervention in resource-limited settings.

**Conclusion:**

Defining the TPP is a key step to align responses across the community to proactively address the potential for clinical failure of artesunate in severe malaria. In the shorter term, artemisinin-based combination therapies should be developed using approved or novel drugs. In the longer term, novel combination treatments should be pursued. Thus, this TPP aims to direct efforts to preserve the efficacy of existing treatments while improving care and outcomes for individuals affected by this life-threatening disease.

## Background

In 2017, Medicines for Malaria Venture (MMV) published a paper identifying gaps in the malaria armamentarium, including drugs to treat severe malaria, and how these gaps might be addressed [1]. This follow-up publication specifically concerns the target product profile (TPP) for new severe malaria therapies, considering recent insights from field observational studies and developments related to drug resistance [2].

Severe malaria is predominantly caused by *Plasmodium falciparum* [3], though *Plasmodium vivax* and *Plasmodium knowlesi* also cause severe disease [4, 5]. An estimated 1% to 3% of uncomplicated malaria cases evolve to severe cases [3]. Risk of severe disease is dependent upon the level of acquired immunity and co-morbidities and mortality risk increases with increasing parasite burden [6]. In areas of high transmission, those most at risk include young children, pregnant women, people living with HIV, and malaria-naïve travellers or migrants [3, 7–9]. In 2022, 95% of the 608,000 estimated global malaria deaths occurred in the WHO Africa region, and 76% of all deaths were in children under five years old [3]. Pregnant women are three times more likely to progress to severe malaria than non-pregnant women owing to immunological changes during pregnancy and *P. falciparum* placental sequestration [8]. Co-infection with HIV and malaria increases the risk of severe malaria in adults and children, particularly if anti-retroviral therapy is not optimized [9]. In regions of low transmission, immunity may not be acquired in childhood leaving adults vulnerable to severe disease.

Severe malaria is a medical emergency that is often the consequence of delayed presentation or diagnosis [10]. Without treatment, mortality from severe malaria approaches 100%, particularly for cerebral malaria with multiorgan dysfunction or metabolic complications [11]. For surviving patients there may be significant morbidity, including severe anaemia and neurological sequelae [6, 11, 12]. However, with prompt effective anti-malarial treatment and supportive care, the mortality rate from severe malaria is reduced to < 5% [13–15].

### Current treatment

The current recommended treatment for severe malaria, including infants and pregnant/lactating women, is intravenous or intramuscular artesunate for at least 24 h and until oral medication can be tolerated, followed by completion of treatment with three days (a full course) of artemisinin-based combination therapy (ACT) [11].

Parenteral artesunate demonstrated high efficacy for the prevention of mortality in two large-scale clinical trials, with superiority over parenteral quinine, the standard-of-care for many decades [13, 14]. Quinine requires a constant rate infusion three times a day or can be administered twice daily by intramuscular route, which is a reliable and potentially a safer route of administration [16]. Adverse events associated with quinine use include hyper-insulinaemic hypoglycaemia and neural, retinal, and auditory toxicity [17, 18]. Thus, parenteral artesunate also has convenience and safety advantages over quinine, as well as reducing the burden for the healthcare professionals [13, 14].

The follow-up ACT is necessary because although artesunate will rapidly reduce parasitaemia, a proportion of parasites will enter induced dormancy [19]. These parasites evade artemisinin, and up to 50% of uncomplicated malaria cases treated with less than six days of artemisinin monotherapy may have recrudescence of parasitaemia within 28 days, with recrudescence rates ~ 80% in severe malaria [19–21]. This dormancy mechanism is distinct from artemisinin partial resistance and can be addressed by combining artemisinin with another anti-malarial drug, as in the case of ACT for uncomplicated malaria, or in severe malaria by following parenteral artesunate with ACT.

Although parenteral artesunate followed by ACT is highly efficacious, severe malaria continues to cause significant mortality because optimal care is not accessible or systematically delivered [22]. As a stop-gap measure to deliver rapid treatment to children in remote areas, a rectal artesunate formulation was developed based on evidence of its ability to reduce mortality in children facing delays of over 6 h in receiving parenteral treatment [23]. Rectal artesunate is only indicated where prompt access to a higher-level facility is unavailable. Severe malaria requires specialist comprehensive supportive care and cannot be managed in the community setting. Thus, the recommendation of the World Health Organization (WHO) is to treat children < 6 years with a single rectal dose of artesunate, with immediate referral to an appropriate facility for further care including intramuscular or intravenous artesunate followed by a full course of ACT [11].

**Fig. 1:**
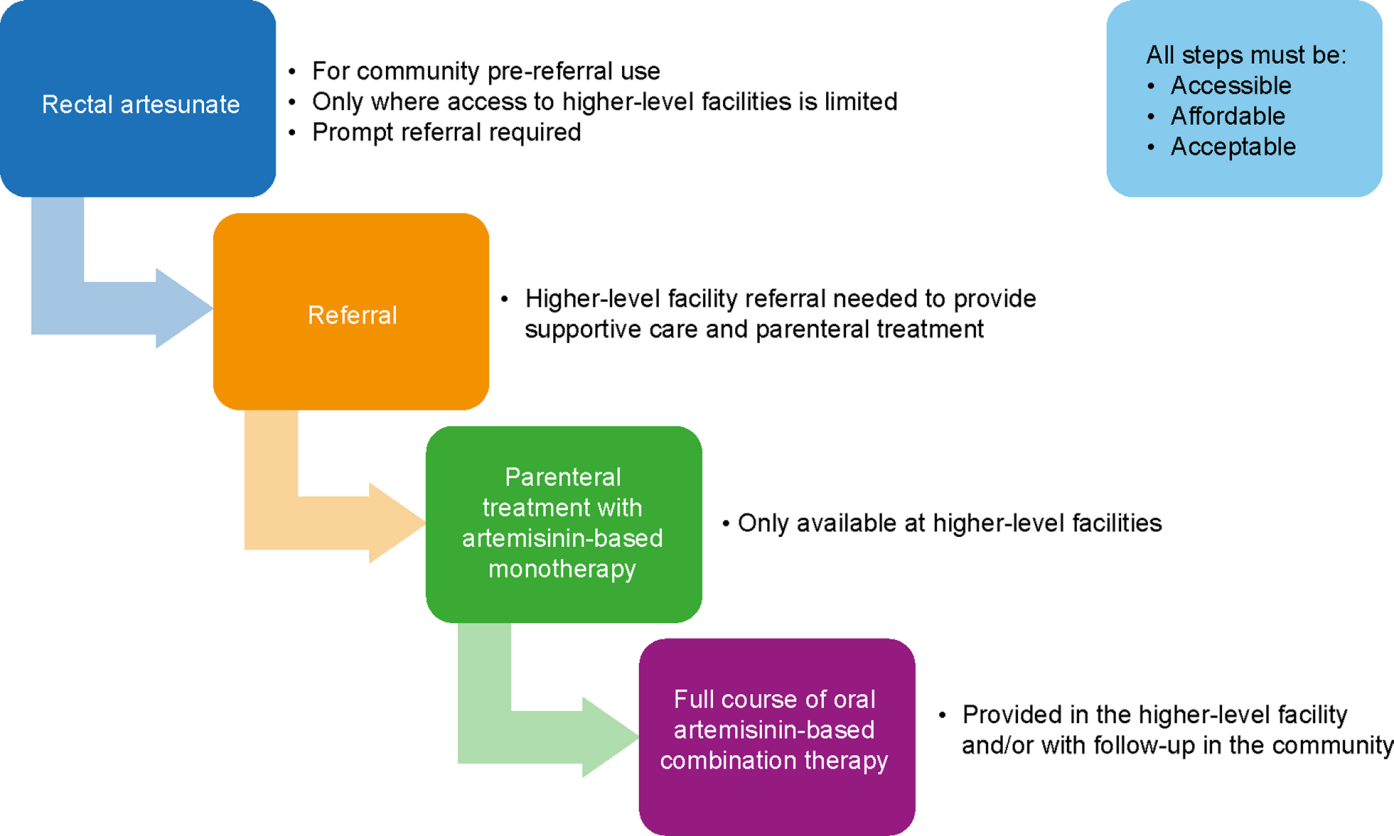
Continuum of care for severe malaria. Rectal artesunate is only indicated where prompt access to a higher-level facility is unavailable. For effective management of severe malaria, patients must complete referral to ensure that appropriate supportive care is available, receive adequate treatment with parenteral artesunate, and complete follow-up oral medication with artemisinin-based combination therapy

The sequential treatment paradigm for severe malaria creates problems in supporting complete adherence to referral and treatment by patients, increasing the potential for poor outcomes and resistance selection (Fig. 1). In malaria endemic countries, maintaining the continuum of care has been challenging, given the insufficiencies of health systems, particularly in remote areas, with added economic burdens on families for completing referrals [22, 24–26]. The number of children who complete referral following pre-referral rectal artesunate is suboptimal, compounded by operational, economic and logistical barriers, and undermines the effectiveness of this intervention [26–28]. Also, following parenteral artesunate, although a full ACT course is crucial to prevent recrudescence, it is often either not prescribed or adherence is incomplete [26–30]. Not only does this risk poor outcomes, but it constitutes artemisinin monotherapy which may promote the selection of drug resistance [26].

## Rationale for the TPP

### Preparedness for impaired artemisinin clinical efficacy

A key concern with the current treatment of severe malaria is that it is highly vulnerable to any reduction in artemisinin clinical efficacy [31]. High-level *P. falciparum* artemisinin resistance has not been observed clinically but can be induced under drug pressure in vivo [32]. Artemisinin partial resistance, which emerged initially in the Greater Mekong sub-region (GMS), is characterized by delayed parasite clearance after therapy [1, 2]. Artesunate has rapid parasiticidal activity and activity against early ring stages [33], deterring maturation to late-stage erythrocytic forms that are most associated with severe malaria pathology [34]. Modelling studies suggest that delayed parasite killing associated with artemisinin partial resistance may affect clinical outcomes for some patients with severe malaria [35]. However, reports of intravenous artesunate failures from the GMS where artemisinin partial resistance is established, but where severe malaria is uncommon, are limited [36, 37]. Nevertheless, WHO guidelines suggest a combination of parenteral artesunate and quinine for severe malaria in regions with artemisinin partial resistance [11], though clinical data are sparse on the feasibility, safety and efficacy of this combination [38].

Non-synonymous mutations in the *P. falciparum kelch* propeller domain (*Pfk13*) have been validated as molecular markers for artemisinin partial resistance [39], and molecular surveillance has shown its independent emergence in Southeast Asia, South America, Oceania and Africa [2]. Mutations known to be associated with delayed parasite clearance have been identified in Ghana, Rwanda, Uganda and Tanzania [3, 40]. There is a clear knowledge gap regarding the clinical impact of artemisinin partial resistance in severe malaria in the context of Africa, and in particular for children under the age of 5 years. In Rwanda, day 3 positivity rates following ACT of uncomplicated malaria were 15% and associated with *Pfk13* R561H. These findings continue to fuel the debate regarding the continued efficacy of artemisinin-based monotherapy for severe malaria in Africa.

### Simplifying the continuum of care

A large implementation study conducted in the Democratic Republic of the Congo, Nigeria, and Uganda pointed to shortcomings in the quality of care for severe malaria [24–26, 41, 42]. These included a lack of referral completion post administration of rectal artesunate and inadequate follow-up with oral ACT, with many patients receiving only rectal and/or parenteral artesunate [24–26, 41, 42]. Although these issues stem from deficiencies in healthcare systems, simplification of the continuum of care, with fast acting drugs that provide rapid cure and prevent recrudescence, should be an objective of drug development.

### Addressing uncertainty in the continuum of care

**Fig. 2:**
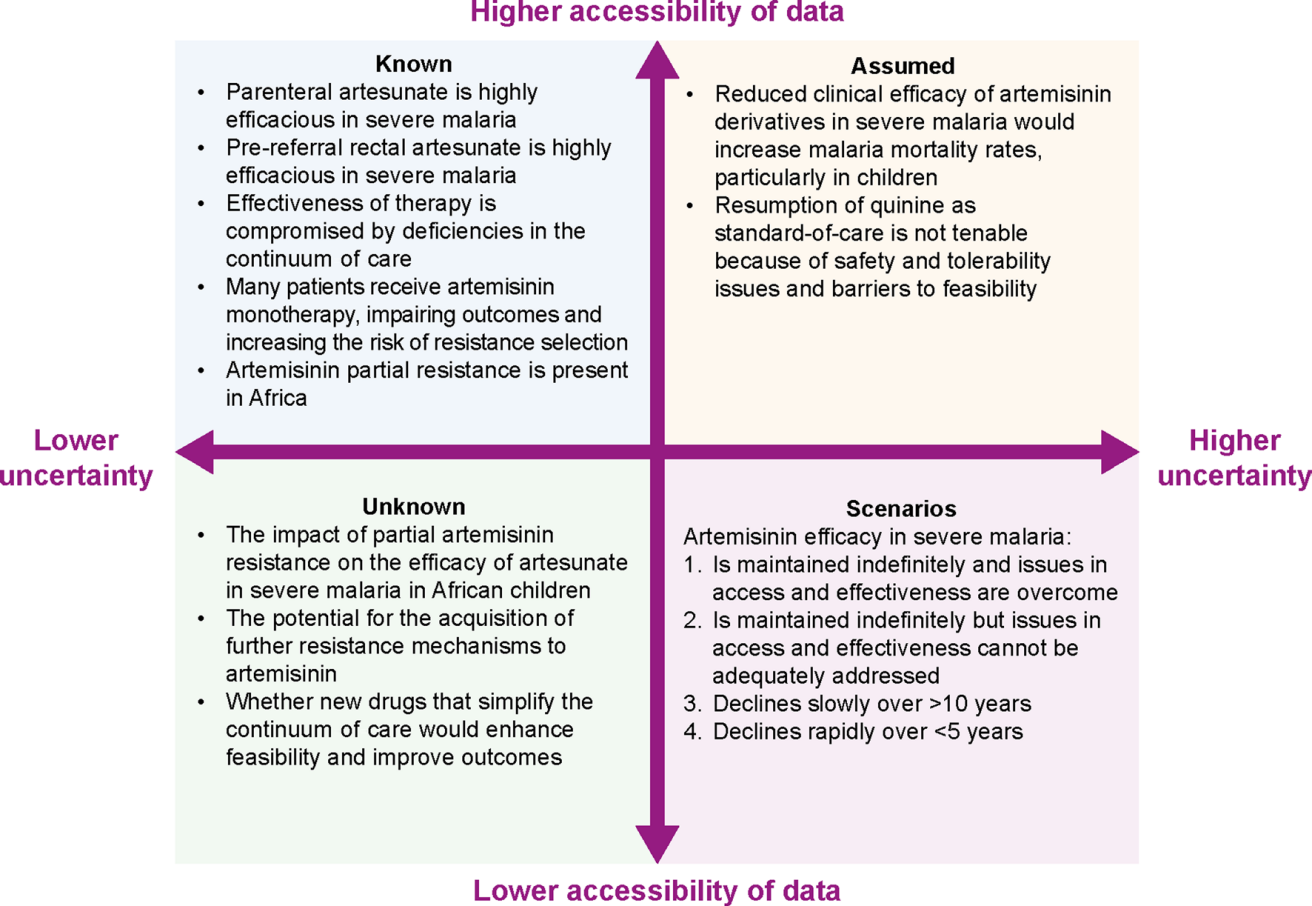
Uncertainty matrix for the treatment of severe malaria with artemisinin derivatives

There is uncertainty regarding the impact of artemisinin partial resistance on outcomes and the potential for further evolution of parasite resistance to artemisinin as well as concerns about the effectiveness of the continuum of care. The status, assumptions, unknowns and scenarios are summarized in Fig. 2. Given the life-threatening nature of the disease and the potentially serious consequences should artemisinin clinical efficacy in severe malaria be compromised, it is prudent to plan for scenarios 2, 3 and 4 by defining the required characteristics for new treatments in a specific TPP for this indication.

## Updating the TPP for new severe malaria therapeutics

### Strategy for new therapeutics

A near-term approach is to add approved anti-malarial compounds as partners to the current parenteral and rectal artesunate-based therapies. This would enable efficacy against *P. falciparum* in the setting of artemisinin partial resistance and mitigate the risk of further selection and dissemination of resistant parasites. Using a partner compound which is already approved for uncomplicated malaria will shorten the development time because pharmacokinetics, safety, tolerability and parasite kinetics are already well understood. However, the risk of multi-drug resistance selection is a concern [3, 43]. There is also the possibility of adding a novel compound to the current parenteral and rectal artesunate-based therapies.

In the longer term, there is a need to develop alternatives to artemisinin and to identify completely new drug combinations. These could be prioritized to proof-of-concept, i.e., demonstration of clinical efficacy in children with severe *P. falciparum* malaria. This would allow rapid advancement and deployment should the clinical efficacy of currently available therapy be compromised. New severe malaria therapeutics should be developed as fixed-dose combinations formulated for parenteral and rectal administration. If physicochemical properties permit, a drug combination should be developed for both parenteral and rectal administration. If this is not possible, different combinations should be developed for parenteral and rectal administration. To reduce the risk of resistance selection, combinations should include two drugs with different mechanisms of action and, ideally, matched pharmacokinetic/pharmacodynamic attributes [44]. There is the possibility that a sufficiently potent parenteral fixed-dose combination could elicit rapid cure, negating the need for oral ACT follow-on treatment.

There are several new compounds in the current anti-malarial portfolio that have potential for development for severe malaria. Given the limited resources available, the TPP will allow efficient prioritization of these compounds. The TPP was prepared after MMV-led consultations with leaders in the research and clinical management of severe malaria and the MMV Expert Scientific Advisory Committee. Table 1 summarizes the elements considered and the relevant targets based on minimal targets and ideal criteria.

**Table 1:** Target product profile for the treatment of severe malaria

Parameter	Minimum essential	Ideal
Indication	• Severe malaria caused by *P. falciparum*	• Severe malaria caused by *P. falciparum* and any *Plasmodium* species or mixed infection^a^
Use population injectable	• Children of all ages OR • Adults and adolescents, including use in pregnancy (all trimesters)	*Same as minimum essential* AND Patients living with HIV and other chronic infections
Use population rectal	• Children of all ages	*Same as minimum essential*
Formulation/presentation	• Formulated to achieve rapid distribution (injectable and/or rectal) • Injectable formulation with minimal, simple reconstitution prior to dosing • Feasible volume like that of injectable artesunate	• Ready to use injectable formulation, in a pre-filled syringe or a blow-fill-seal device • Injection volume lower than that of injectable artesunate
Dosing regimen for injectable	• 2–3 times daily administration for the first 24 h and until an oral uncomplicated malaria treatment can be administered • Dosing adjusted for patient weight	• Once- or twice-daily administration (without the need for oral follow-up treatment)
Dosing regimen for rectal for pre-referral	• Single dose	*Same as minimum essential*
Duration of treatment	• Equivalent to current therapy including 3-day follow-up with oral uncomplicated malaria treatment	• Reduced treatment duration without the need for oral follow-up treatment
Continuum of care	• Equivalent to current recommendations (Fig. 1)	• Simplification by eliminating any step, e.g., rapid cure without the need for oral follow-up treatment
Parasite stage of action	• At least one component with good potency against ring-stage parasites	• Both components with good potency against ring-stage parasites and other erythrocytic stages with or without activity against gametocytes
Parasite clearance kinetics	• At least one component with immediate onset of action • At least one component with parasite clearance kinetics equivalent to artesunate	• Both components with immediate onset of action • Both components with parasite clearance kinetics equivalent to artesunate • One component with parasite clearance kinetics superior to artesunate
Susceptibility to resistance	• Active against known drug-resistant clinical isolates • Low propensity of the combination for resistance selection • Resistance marker known and no cross resistance with pre-existing resistance mutations	• Active against known drug-resistant clinical isolates and laboratory generated drug-resistant parasites • No selection of resistance mutations with the combination
Safety	• No severe drug-related adverse effects • No need for post-treatment cardiac monitoring • Non-inferior to artesunate for risk of delayed haemolysis	• No risk of delayed drug-induced haemolysis
Drug-drug interactions	• Minimal drug interactions manageable with dose adjustments	• No clinically significant drug interactions
Shelf-life of the drug product	• ≥ 2 years at ICH zone IVa/IVb conditions^b^	• ≥ 3 years at ICH zone IVa/IVb conditions^b^
Cost of treatment course	• Price comparable to the standard-of-care^c^	• Price equivalent or lower than the standard-of-care^c^

^a^Activity against non-falciparum species would be demonstrated in vitro and would not be evaluated specifically in clinical studies of severe malaria

^b^ICH zone IVa is hot/humid zone (30 °C and 65% relative humidity) and IVb is hot/higher humidity zone (30 °C and 75% relative humidity) defined according to the International Council for Harmonization of Technical Requirements for Pharmaceuticals for Human Use (ICH) climate stability zone criteria [45]

^c^Artesunate reference prices as per the Global Fund to Fight AIDS, Tuberculosis, and Malaria in Q4 2023 were: artesunate powder for solution for injection 30 mg $1.36, 60 mg $1.37, and 120 mg $2.41, and rectal artesunate 100 mg (2 rectal capsules) $0.70, artesunate 100 mg suppository (2 pack) $0.70 [46]. However, given that most of the cost of management of severe malaria relates to the hospitalization, then higher prices may be justified in cases of severe malaria which cannot be managed with artesunate

### Indication and population

The main purpose of any new severe malaria product is the treatment of *P. falciparum* infection in children under 6 years of age. Severe malaria is relatively uncommon in African adults but there are populations who are at risk for severe malaria, i.e., those living with HIV or other chronic infections and malaria-naïve travellers or migrants [3, 7–9]. For example, people living with HIV or other chronic infections, such as tuberculosis, may be taking medications and ideally new anti-malarial drugs would not be contraindicated in these populations owing to drug–drug interactions.

Furthermore, pregnant women are at increased risk of a severe form of malaria, and severe anaemia [47]. The inclusion of pregnant women in the target population is not only a medical need but an equity consideration [48]. Historically, pregnant women have been excluded from the drug development process, necessitating the post-registration collection of data on medication safety during pregnancy through resource-intensive studies such as pregnancy exposure registries, case–control studies, and surveillance. In malaria-endemic regions, this could be challenging due to weak pharmacovigilance systems, irregular patient follow-up, and limited resources. Consequently, there have been significant delays in the availability of efficacy and safety data during pregnancy, impeding equitable access to new anti-malarial drugs by pregnant women. There have been growing calls in recent years for a paradigm shift to integrate the evaluation of drugs in pregnancy much earlier in the development process to close the time lag.

The TPP proposes intentional and systematic data collection from the pre-registration stages of development through post-licensure to inform on the benefit:risk balance of new treatments for severe malaria during pregnancy. Initially, this involves efficient in vitro screening and prioritization of lead compounds for non-teratogenic profiles. Developmental reproductive toxicity studies in animals can then be conducted early in the process to allocate resources for clinical studies towards those drugs with the greatest potential for use in pregnancy. Physiologically-based pharmacokinetic modelling is a valuable tool to anticipate necessary adjustments for drug dosing throughout pregnancy [49–53]. Phase 1 clinical pharmacology trials for pregnant women should commence concurrently with phase 3 trials for the non-pregnant population [48]. Given the potential lethality of the disease, the benefit:risk profile in severe malaria is weighted to ensure maternal survival unless it is known that a drug presents a specific risk to the fetus.

While it is anticipated that the initial labelling for a new treatment may not specifically include pregnant women, the TPP considers this population from the outset of the drug development programme. This proactive approach also protects women and adolescent girls of childbearing potential who might be treated for severe malaria before their pregnancy is detected. Thus, the TPP stipulates that different drug classes may be developed for children and the adult/adolescent populations to reflect the different reproductive toxicity profile requirements.

### Formulation and presentation

Patients with severe malaria are rarely able to take oral medication and need parenteral anti-malarial therapy to quickly reduce/clear parasitaemia. Drug combinations should be co-formulated and ideally offer a reduced dosing frequency compared with current therapy. Artesunate for intravenous or intramuscular injection requires reconstitution, the solution cannot be prepared in advance and the procedure is vulnerable to human error. New injectable formulations should require minimum reconstitution prior to dosing and should be stable in high temperature conditions. A ready-to-use co-formulation would be convenient, and a pre-filled syringe is an ideal drug product, although new low-cost technologies in this area are urgently needed. A rectal formulation will have specific physiological and pharmaceutical requirements [54], and should be stable long term under high temperature and high humidity conditions with non-refrigerated transport.

### Simplification of the continuum of care

An area which was not explored in previous TPPs was the potential for new drugs for severe malaria to address gaps that currently exist in the artemisinin-based continuum of care (Fig. 1) [1, 55]. Ideally, parenteral administration should be sufficient for clinical and parasitological efficacy leading to a cure, without the need for an oral treatment follow up. Where patients do not have access within a few hours to well-equipped healthcare facilities, there is a role for a pre-referral intervention. Combination therapy would overcome the issues of inadequate referral increasing the risk of resistance selection, as is the case for rectal artesunate monotherapy. However, it may leave the patient vulnerable to recrudescence. Thus, a highly effective anti-malarial that could provide rapid cure or which has a longer duration of action than artesunate could avoid poor outcomes caused by referral delay. However, it should be stressed that referral to hospital is necessary to adequately manage the manifestations of severe malaria.

### Parasite stage of action and clearance kinetics

Severe malaria usually has high parasitaemia and rapid progression. Therefore, treatments must be highly potent, with rapid onset of antiparasitic activity to clear the infection as quickly as possible and prevent complications. Compounds which are active against the early ring stage have a higher potential for rapid action, as observed for artemisinin, cipargamin (KAE609) and SJ733 [56, 57]. Also, as the pathology of severe malaria is driven by the sequestration of late stage intraerythrocytic forms in tissues and organs [34], drugs which can interrupt progression to these stages are likely to generate better outcomes, as seen with artesunate, which is active against ring-stage parasites [13, 14]. Ideally, drugs for severe malaria would kill all erythrocytic stages with a low nanomolar potency, and with potential to support a low dose in patients. Dose is not only a factor for clinical management but is often a considerable driver of the eventual cost of the product.

### Potential for drug resistance

Drugs for use in severe malaria must retain full activity against all existing clinically resistant strains of parasite and should ideally have a particularly low propensity for the selection of resistance in *P. falciparum*. A key concept is that a combination of drugs with different mechanisms of action can protect each other from resistance development. Random non-synonymous mutations occur at a frequency of around 1 to 5 per 10^9^ for blood-stage parasites [58]. In severe malaria, parasitaemia can be as high as 1.9 million parasites/µL (10^12^ parasites per human infection), increasing the statistical risk of resistant mutant selection [59]. Similarly, gene amplifications that improve survival will be more often selected at higher parasitaemia under drug pressure [60]. Although drug resistance usually manifests as a loss of efficacy during the erythrocytic cycle, artemisinin partial resistance is observed as a delay in parasite clearance [61]. Similar resistance mechanisms could potentially emerge to compounds with similar parasite kinetics. Thus, for any new fast-acting compound used against severe malaria, it will be important to assess the potential for both conventional resistance acquisition and changes in parasite killing kinetics, and it will be important to consider risks in the context of a combination regimen.

### Safety and tolerability

New drug combinations for severe malaria will need to have a safety and tolerability profile at least as good as that of artesunate. The main safety issue with artesunate is delayed haemolytic anaemia, which is observed particularly among non-immune travellers and young children with high parasitaemia [62, 63]. Artesunate has a dose limiting adverse event of neutropenia which precludes the use of higher doses to overcome any clinical impact of artemisinin partial resistance [64, 65]. Ideally, new drugs would not have these issues. Artesunate can be given throughout pregnancy and evaluating new drugs in pregnancy requires assessment of developmental and reproductive toxicity risk before clinical data can be captured via post-marketing surveillance, which can take some considerable time [66].

New therapies should ideally not have drug–drug interactions that affect the efficacy or safety of anti-malarial therapy or essential concomitant therapies, though dose adjustments for concomitant therapies could be acceptable if necessary. This is particularly important given that the management of severe malaria involves addressing diverse symptoms, which require additional therapies such as diazepam, lorazepam, midazolam or intramuscular paraldehyde for convulsions, analgesics and antipyretics for pain and fever, and broad-spectrum antibiotics for bacterial co-infections [11]. Additionally, the patient could be receiving medications for any pre-existing conditions or infections, including HIV and tuberculosis or other non-communicable co-morbidities.

### Logistical and cost considerations

A key area for improvement versus current therapies is thermostability. Rectal artesunate capsules have impaired stability above 30 °C and particularly above 40 °C and 75% relative humidity, meaning that stock does not generally exceed six months, increasing the risk of stockouts [67]. New drugs should be developed with sufficient shelf-life in field conditions to ensure stocks are available when needed. Additionally, the drug products should allow easy, non-refrigerated transport. The cost of goods for any new treatments for severe malaria will need to be consistent with a purchase price that is not greater than currently available treatments. However, drug purchase price represents a small cost relative to the overall implementation costs of the severe malaria continuum of care, particularly when patients are hospitalized or when treatment fails [68]. Thus, a more costly drug, which decreases the duration of hospitalization or prevents recrudescence without the need for follow-up oral therapy could be more cost-effective than one which must be implemented within the current continuum of care paradigm.

## Severe malaria pipeline

**Fig. 3:**
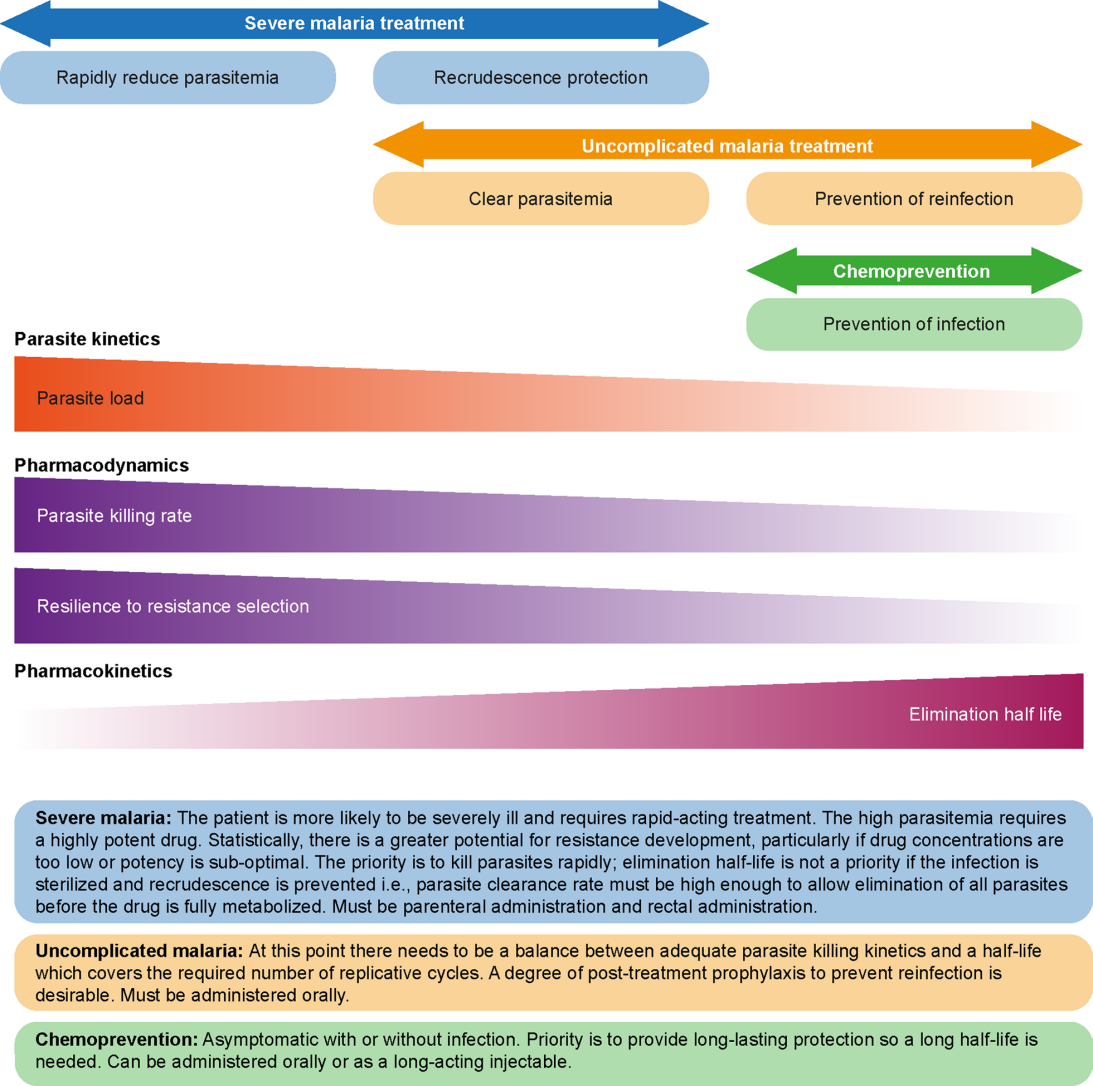
Drug development focus across the spectrum of malaria indications

In earlier proposals for TPPs, it was envisaged that new treatments for severe malaria would be developed from drugs already approved for the treatment of uncomplicated malaria [1, 55]. Although there is some overlap in the required characteristics of drugs for malaria, there are specific features of severe malaria that warrant a tailored approach (Fig. 3). Additionally, the risk:benefit profile for a new drug in severe malaria, which is life threatening, will be different to that for uncomplicated malaria, where the aim is to prevent disease progression and deliver cures, and chemoprevention in an asymptomatic or uninfected population.

Key considerations for drug development of new combinations for severe malaria are that they should have immediate onset, be fast-acting and are available at a dose which allows formulation for parenteral administration (preferably by intravenous and intramuscular routes) and/or for rectal administration. They should not have a negative effect on the host response to infection, either via innate immunity or the significant physiological consequences of severe malaria. Ideally, both components of a combination drug should have a low propensity for resistance selection, different mechanisms of action and a half-life that is matched to protect against resistance selection. Rapid anti-malarial activity is assessed using the parasite clearance half-life (PCT_1/2_), which is the time required for the parasite density to be reduced by 50% along the log-linear portion of the normalized parasite clearance curve [69]. Anti-malarial activity can also be expressed as a parasite reduction ratio (Log PRR), which is a log_10_ drop of viable parasites within 48 h in vitro [70]. Thus, a smaller PCT_1/2_ and a larger log PRR indicates faster clearance of parasites from the blood. Additionally, the PRR and PCT_1/2_ can be evaluated in animal models and in volunteer infection studies in humans. However, findings may differ from the in vitro results as it is not possible to differentiate between viable and non-viable circulating parasites. Another possible reason for differences between situations in patients versus in vitro is that, in patients, PCT_1/2_ is the result of parasite killing by the drug and parasite clearance by the host while in vitro PRR reflects only the parasite killing property of the drug.

The potential for resistance selection can be evaluated systematically by characterizing druggable molecular targets and associated resistance mechanisms [71, 72]. A key metric is the minimum inoculum for resistance (MIR) [72], with higher values associated with a lower risk of resistance selection [58]. The ideal in early drug development is a compound with logMIR > 9, meaning that no stable resistant mutants are selected in laboratory conditions for up to 60 days. However, the overall resistance selection potential of the drug combination is key rather than that of the individual components. Thus, a drug with some propensity for resistance selection could still have utility if combination with a suitable partner drug resulted in an irresistible combination therapy.

For developing an artemisinin-based combination therapy for severe malaria, the most advanced partners are drugs which have been already approved for the treatment of uncomplicated malaria. These include amodiaquine, lumefantrine, mefloquine, piperaquine, pyronaridine, chloroquine, and quinine (Table 2). An assessment of partner drug suitability following the guidance of the TPP is required.

**Table 2:**
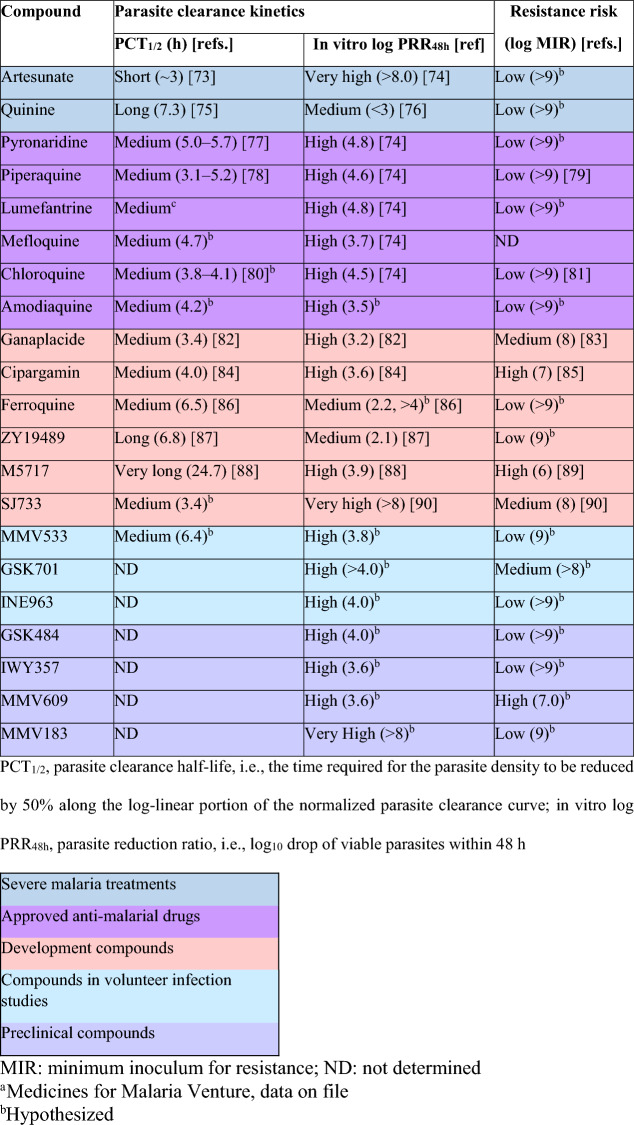
Comparison of key attributes of registered anti-malarial drugs and potential molecules

**Fig. 4:**
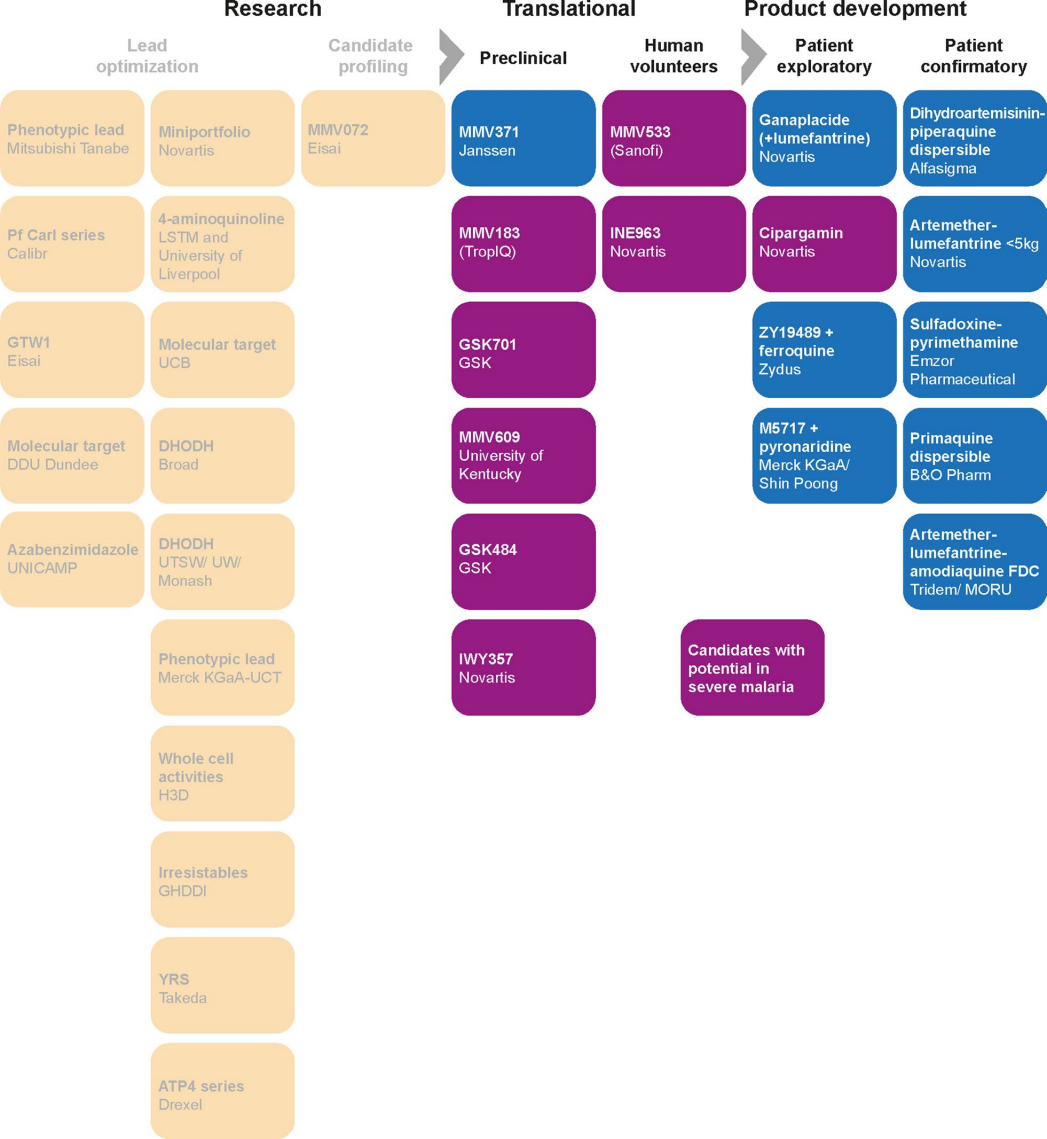
Research and development pipeline, showing potential molecules for severe malaria

When considering new compounds that could be either combined with artesunate or developed into novel drug combinations, there are several molecules in the current development pipeline with characteristics indicating potential for the treatment of severe malaria (Table 2, Fig. 4) [73–90]. The most advanced candidate is cipargamin, a novel spiroindolone anti-malarial targeting PfATP4 currently in phase 2 development by Novartis [56, 91–93]. MMV609 (MMV1793609) is also a PfATP4 inhibitor and has similar parasite killing kinetics to cipargamin in preclinical models [94]. Another PfATP4 inhibitor, the spiroindolone SJ733, had rapid parasite killing in a human volunteer infection study [95]. MMV533 (MMV688533) is an acylguanidine that was developed following a whole-cell screen, and which has rapid parasite killing and a low potential for resistance [96]. Additional possibilities include INE963 (NVP-INE963, MMV1582617) which has a novel but unknown mode of action [97], and the acetylCoA synthetase inhibitors MMV183 (MMV693183) [98, 99], and GSK701 (MMV1582367) [100]. Additionally, GSK484 (MMV1793192) and IWY357, both of which have unknown modes of action, have rapid parasite killing and a low potential for resistance selection (Table 2). Three additional molecules, ganaplacide (KAF156), ZY19489 and ferroquine, which are currently being investigated for uncomplicated malaria could also be considered for severe malaria (Fig. 4) [101–103].

The novel *Plasmodium* eukaryotic translation elongation factor 2 inhibitor M5717 (formerly DDD107498) blocks parasite modification of host red blood cells (RBCs) by preventing synthesis of new exported proteins [104]. M5717 presents some interesting perspectives on anti-malarial drug development for severe malaria. The drug has a long half-life but demonstrates a distinct delay in parasite clearance in both mouse and human studies [88, 105, 106]. This delay has been recently explained by an M5717-induced decrease in the rigidity of infected red blood cells, reducing splenic clearance while inhibiting sequestration into tissues [104]. The clinical implications of these findings for severe malaria require further investigation but indicate that for drugs with novel mechanisms of action, it may be necessary to consider attributes beyond the classical measures of PCT_1/2_ and PRR. Interestingly, pyronaridine potentiated the pre-erythrocytic activity of M5717 in the *Plasmodium berghei* mouse model, highlighting that combination therapies could have additional benefits beyond their individual components [107].

## Considerations for drug development in severe malaria

The translational pathway for compounds for severe malaria relies heavily on a deep understanding of the biology of the compound, parasite killing kinetics, and pharmacokinetics. Preclinical models of cerebral malaria are still over-reliant on a *P. berghei* (ANKA strain) infection in C57BL/6J adult mice, which is not an accurate model for *P. falciparum* infection. There is no validated animal model that can predict clinical efficacy in severe malaria, though the HuSICD mouse is useful for predicting parasite reduction kinetics. Human volunteer studies are therefore important to demonstrate the safety, tolerability, and the feasibility of drug administration by the intravenous and/or intramuscular route. Volunteer infection studies involving controlled infections can provide an early read out on potential efficacy. Although severe malaria cannot be directly investigated in these models, blood-stage parasite growth dynamics can be effectively characterized [84, 108, 109]. Using this information and human pharmacokinetic data, it should be possible to estimate the efficacy of novel agents in severe malaria using pharmacokinetic/pharmacodynamic modelling, benchmarked against agents with known efficacy in severe malaria. Also, it may be possible to identify biomarkers that are indicative of clinical efficacy in severe malaria [12]. Following investigations in adults there is potential to bridge pharmacokinetic and pharmacodynamic data to children using physiologically-based pharmacokinetic modelling [110]. However, even using this approach, children may not respond in the same way to drugs as adults [111].

Clinical outcome in severe malaria studies has typically been evaluated in terms of mortality [13, 14]. However, any new therapy would need to demonstrate non-inferiority to artesunate and because artesunate has high efficacy, the required sample size may not be feasible [14]. Thus, alternative clinical endpoints are under investigation with the aim of predicting efficacy in severe malaria using a lower sample size. For example, improvements in lactate concentrations assessed at 8 to 12 h after admission have been suggested as a surrogate endpoint for the evaluation of anti-malarial drugs for severe malaria [112]. The on-going phase 2 KARISMA study (ClinicalTrials.gov identifier NCT04675931), evaluating cipargamin in adult and paediatric patients with severe *P. falciparum* malaria, uses the percentage of participants achieving a ≥ 90% reduction in *P. falciparum* at 12 h post-dose as the primary outcome measure to determine the most appropriate dose to take into phase 3 studies. The study will evaluate clinical success using a novel composite clinical endpoint based on survival, parasite clearance and the absence of key complications of severe malaria. It is expected that results of the KARISMA study and other similar investigations will inform more efficient clinical drug development strategies for severe malaria.

## Conclusions

Severe malaria is a serious medical emergency and rapid effective treatment is necessary to save lives and prevent complications. The dependence on artemisinin monotherapy for the initial treatment of this disease is a concerning vulnerability that should be addressed given the risk of artemisinin partial resistance. Additionally, a monotherapy treatment paradigm with inadequate subsequent case management increases the risk of recrudescence and the potential for the selection of artemisinin partial resistance.

The proposed TPP for new therapies is a step towards preparedness to address threats to the currently efficacious treatment of severe malaria. The following strategy is proposed. In the short term, parenteral and/or rectal formulations of identified partner drugs should be developed to be co-administered with artesunate to protect against the selection of artemisinin partial resistance and the potential emergence of further resistance mechanisms to artemisinin. The most advanced partners are those which are already approved for the treatment of uncomplicated malaria, though new drug candidates could also be considered. In concert, novel non-artemisinin combination treatments should be developed (preferably fixed-dose combinations), at least through phase 2, allowing rapid advancement to the clinic should the efficacy of existing therapies become compromised. There are several potential candidate molecules in the malaria portfolio which have attributes consistent with the TPP requirements. New treatments should be available as parenteral and/or rectal formulations. There is also the possibility to simplify the continuum of care by providing longer-acting combinations for severe malaria, negating the need for follow-up oral medication.

Pursuing new drug development and combining therapies proactively prepares for any attenuation of artemisinin clinical efficacy in severe malaria. These efforts will also extend the longevity of existing treatments while improving care and outcomes for individuals affected by this life-threatening disease.

## Data Availability

No datasets were generated or analysed during the current study.
